# Sequential laxative-probiotic usage for treatment of irritable bowel syndrome: a novel method inspired by mathematical modelling of the microbiome

**DOI:** 10.1038/s41598-020-75225-z

**Published:** 2020-11-09

**Authors:** Ming Li, Ri Xu, Yan-qing Li

**Affiliations:** Department of Gastroenterology, Qilu Hospital of Shandong University, 107 Wenhuaxi Road, Jinan, 250012 China

**Keywords:** Irritable bowel syndrome, Applied mathematics

## Abstract

The gut microbiome plays an important role in human health. However, its response to external intervention is complex. A previous study showed that the response to *Clostridium butyricum* (CB) treatment of irritable bowel syndrome (IBS) is heterogeneous. We proposed that mathematical model simulation of the microbiota may help to optimize the management of IBS-associated microbiota. In this study, a novel mathematical non-extinction and defecation normalized (NEDN) model was generated for stable simulation of the dynamic nature of gut microbiota. In silico simulation revealed that a laxative may create a favourable opportunity for Clostridium cluster XIVa to shift the microbiota. An explorative clinical trial was conducted to compare three CB regimens in an IBS cohort: laxative, interval of 2 weeks and CB administration for 2 weeks (L2P); laxative immediately followed by CB administration (LP) for 2 weeks; and CB administration for 2 weeks (P). The LP regimen optimally relieved the IBS symptoms and shifted the microbiota closer to those of the healthy subjects during 2 weeks of CB intake. These results indicate that integration of biological/mathematical approaches and clinical scenarios is a promising method for management of microbiota. Additionally, the optimal effect of sequential laxative-CB usage for IBS treatment warrants further validation.

Clinical trial registration numbers: NCT02254629.

Date of registration: October 2, 2014.

## Introduction

The intestinal microbiota has key functions in human health. Malfunction of the gut microbiota, often termed “dysbiosis”, has been shown to participate in multiple diseases^1,2^. Thus, the microbiota represents a promising intervention target^3^. However, the intestinal microbiota is inherently dynamic^4,5^, and the laws of its behaviour are, unfortunately, almost completely unknown^6,7^. This hinders the precise manipulation of the gut microbiota. Some of the microbiota-targeted interventions do not work^8^ and may even result in undesired effects^9^. Given the complexity of the microbiota, it is not feasible to permute and test all parameters in the animal models or clinical trials. Some of the considerations may be applicable for probiotic usage in irritable bowel syndrome (IBS), a severe functional intestinal disorder. The optimal dosage, treatment duration and combination of bacterial strains remain to be determined^10^.

Mathematical models can describe and predict ecosystems. A previous study suggested that the composition of the human faecal microbiome at a given time point is a major factor defining microbiome composition^11^. We hypothesized that mathematical modelling can streamline the clinical management of the gut microbiota. Mathematical models have been used to describe a wild-type ecosystem, and some of the models have been directly implemented in the gut^12,13^. However, the gut microbiome is fundamentally different from the wild-type ecosystems in many aspects. For example, humans defecate, and the intestinal microbes can gain new niches and grow with less constraint after each defecation. Thus, a new suitable model for the gut microbiota should be developed. In this study, we propose a new non-extinction and defecation normalized (NEDN) model; the parameters of the model were fit based on the previously published time-series data. The behaviour of the microbiota and its responses to various external disturbances were simulated. Laxative-treated microbiota at low density is more easily modified by probiotics. Furthermore, a preliminary clinical trial was conducted to demonstrate that administration of *Clostridium butyricum* to IBS patients immediately after the laxative is more effective than uncoupled usage of the agents.

## Results

### The NEDN model of the gut microbiota

**Figure 1:**
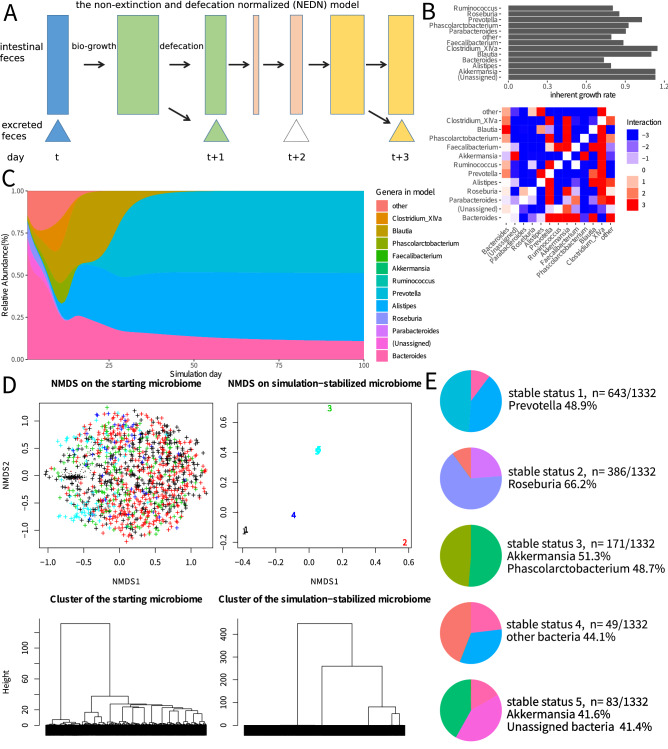
The non-extinction and defecation-normalized (NEDN) model of the faecal microbiome and its dynamic properties. (**A**) The schematic diagram of the NEDN model. (**B**) The inherent growth rate (vector α) and the inter-genus interaction (matrix β) for the genera in the NEDN model. The microbiome data used to fit this model are publicly available data from Caporaso et al.^15^. (**C**) Iterative simulation of the NEDN model of the faecal microbiome showing a self-stabilization trend. (**D**) The NMDS (upper left) and Bray distance-based cluster (lower left) analysis of 1,000 random simulated microbiomes ( +) and 332 experimental microbiomes (dots) in the previously published data. Both types of microbiome were simulated by the NEDN model, and their stable composition was analysed in a similar manner (NMDS, upper right; cluster, lower right). The starting microbiome and the corresponding simulation-stabilized microbiome are shown in the same colour in the NMDS analysis. (**E**) The compositions of the five stable microbiomes. These five stable statuses correspond to the numbers in the NMDS plot of the stable microbiome (**D**, upper right), and the genus colour is identical to that in (**C**).

A new NEDN model was developed to simulate the dynamic behaviour of the gut microbiome. The NEDN model was evolved from the generalized Lotka–Volterra (gLV) model^14^ and was modified to fit the nature of the gut microbiome and the pyrosequencing data. The NEDN model assesses the daily data and reads the relative abundance data of the bacterial community. The NEDN model assumes that every day the microbiome moves through two steps, including the biological growth and possible defecation steps (Fig. 1A). In the biological growth step, each genus grows according to its inherent growth rate α and is influenced by all other genera (interaction described by matrix β). If the predicted abundance of any genus becomes lower than the minimal limit 1*10^–6^, it will be replaced by 1*10^–6^. Thus, all genera enrolled in the model are never eliminated. This assumption is rational because the gut has multiple niches for microbes, e.g., the mucosal microbiota. Although the abundance of some genera may fall below the detection limit of pyrosequencing during the compositional fluctuations, these genera are unlikely to die out. This assumption ensures that all genera enrolled in the model are never eliminated thus generating a non-extinction model. In the defecation step, if total abundance is higher than 1, it is normalized to 1, and the abundance of all genera is reduced by the same factor. Thus, gut bacteria may grow constantly, while their total mass within the host may remain unchanged because excessive biomass is discharged through defecation. The equations of the NEDN model are as follows:

- The biological growth step:
  $$\overrightarrow{{x}_{bio-growth}}=max\{{10}^{-6},\overrightarrow{{x}_{t,faecal}}\cdot [1+\overrightarrow{\alpha }\cdot (1-\overrightarrow{{x}_{t,faecal}})+\overrightarrow{{x}_{t,faecal}}\times \beta ]\}$$
- The possible defecation step:
  $$\overrightarrow{{x}_{t+1,faecal}}=\frac{\overrightarrow{{x}_{bio-growth}}}{max(1,\sum \overrightarrow{{x}_{bio-growth}})}$$

where the vector *x*_t,faecal_ represents the relative abundance of modelled genera in the current faecal sample. The vector α and matrix β are the inherent growth rate and interaction matrix, respectively, similar to those in the classic gLV model^14^. The vector *x*_*bio-growth*_ is the presumed abundance of each genus before the next day defecation. The vector *x*_t+1,faecal_ represents the relative abundance of the modelled genera on the next day.

Initially, the NEDN model was fit to previously published dense time-series data^15^. The inherent growth rate α and interaction matrix β of the enrolled genera were calculated and are shown in Fig. 1B. The novel NEDN model is a better fit of the dynamic changes of gut microbiome than the classical gLV model. The NEDN model has a lower cumulative deviation than that of the gLV model when fitted to the same dataset (L value 10.17 vs 11.19 under the same α and β constraints). Importantly, the inherent growth rate in the NEDN model is positive in contrast to the all-negative growth rate in the gLV model (Fig S1A). Additionally, the negative growth rate contradicts the proliferating characteristics of bacteria. The model can predict the day + 1 to day + 3 microbiome based on the day 0 data. The error of the gLV model is rapidly accumulated; however, the error of the NEDN model remains stable (Fig S2 A-C). Thus, the NEDN model may be better tailored to the gut microbiome than the classical gLV model.

### In silico simulation of the microbial dynamics

Next, the dynamic behaviour of the microbiome was investigated. The microbiome was iteratively predicted based on the starting microbial composition in the time-series data^15^. A typical dynamic behaviour is shown in Fig. 1C, where the microbiome evolves and stabilizes itself, reaching a “stable status”. Then, all possible stable statuses of the microbiome were identified. A set of the starting microbiome was generated to include: (1) the real microbiome based on 332 faecal samples obtained in the previous study^15^; (2) a random microbiome generated by 1000 samples of 13-dimensional random Dirichlet distribution of the composition. The non-metric multidimensional scaling (NMDS) analysis showed that the random microbiome (Fig. 1D, plotted in +) encompasses the real microbiome (Fig. 1D, plotted in dots). After iterative simulation, all 1332 starting microbiomes reached a stable status. The NMDS and cluster analysis showed that the microbiomes converged at five stable statuses (Fig. 1D). The composition of the stable microbiome is plotted in Fig. 1E. The most common type was Prevotella-dominant (48.9%), accounting for 643 of the 1332 starting microbiomes. These simulations indicate that the microbiome is dynamic and tends to stabilize itself to a few stable statuses.

**Figure 2:**
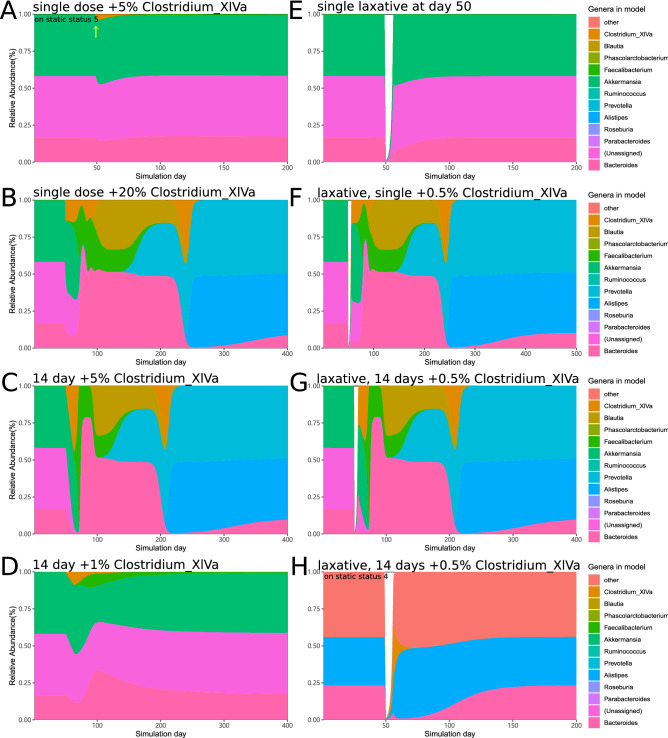
In silico intervention changes the microbiome based on the NEDN model. The simulation starts with the stable status 5 as the initial microbiome (except for the bottom left that starts with stable status 4). Two types of in silico intervention are included: (1) probiotic-like that increases the abundance of a given genus and (2) laxative-like that reduces the abundance of all genera to 1% of their original value. The probiotic-like intervention is administered either once at day 50 or daily from day 51 to day 64. The laxative-like intervention is administered at day 50 (if applicable). The time frame is extended until a microbiome reaches a stable status.

Then, we determined how the gut microbiome dynamically responds to artificial interventions. The dynamics of the microbiome were simulated in silico under two types of artificial perturbations: (1) probiotic-like intervention by Clostridium cluster XIVa that was added at variable frequency and doses; and (2) laxative-like treatment to reduce the whole microbial population to 1% of the original population (Fig. 2). Due to lack of relevant data and simplification of the longitudinal gastrointestinal tract assumed to be a single chamber, the intensity of laxative treatment was artificially estimated rather than obtained from an actual experiment. The statuses 5 and 4 are minor statuses with lower frequency (83 and 49, respectively) than statuses 1–3 (643, 386 and 171, respectively). The status 5 has substantial proportion of unassigned bacteria (41.6% Akkermansia and 41.4% unassigned bacteria, Fig. 1E). Thus, status 5 may represent unhealthier microbiome that requires an intervention. The in silico intervention was started from day 50 after status 5 became stable.

Initially, we determined whether a single dose of a single genus of a bacterium can influence the gut microbiome in a dose-dependent manner. Addition of Clostridium cluster XIVa at a single dose equivalent to 5% of the gut microbes caused only short-term minor disturbance of the gut microbiome. (Fig. 2A). A single dose of Clostridium cluster XIVa equivalent to 20% of the microbiome (Fig. 2B), which is an unreasonably high dose for clinical use, boosted the abundance of Bacteroides for a period over 100 days, and the microbiome finally stabilized at the stable Prevotella-dominant status 1. Thus, the stable and balanced microbial composition is self-stabilizing and is refractory to external disturbances. Then, daily + 5% Clostridium cluster XIVa was administered for 14 consecutive days, and the microbiome shifted to the Prevotella-dominant status 1 after a Bacteroides-dominant period (Fig. 2C); however, similar administration of + 1% Clostridium cluster XIVa failed to shift the microbiome (Fig. 2D). These data suggest that balanced gut microbiome is difficult to change by addition of a single dose of a single genus of a bacterium. The repeated administration of a single genus of a probiotic is more efficient at shifting the microbiome, and the effect is dose-dependent.

A laxative-like treatment was introduced as a heavy disturbing factor presumed to reduce the intestinal bacterial population to 1% of the original state. Simulation of the laxative effects indicated that the microbiome can restore the original composition within 20 days (Fig. 2E). A considerably lower single dose of 0.5% of Clostridium cluster XIVa added on the next day after the laxative was able to shift the microbiota to the Bacteroides-dominated type and then to the Prevotella-dominated stable status (Fig. 2F). Treatment with + 0.5% Clostridium cluster XIVa for 2 weeks had a similar effect and shifted the microbiome from the static status 5 (Fig. 2G). The strength of simulated laxative treatment was presumed to reduce the microbiota to 1% of its original population. In additional simulations, weaker strength of 5% or 10% did not promote a shift in the microbiota. Excessive strength of approximately 0.1% resulted in an easier shift of the microbiome; however, this treatment was able to cause a microbiome shift on its own (data not shown). Thus, the strength of 1% was adequate to represent the effect of laxative treatment. These data suggest that laxatives may create a favourable opportunity for a single-dose probiotic to modulate the microbiota. The same laxative-Clostridium regimen used in the static status 4 failed to shift the microbiota (Fig. 2H). The key points of the microbiota simulation are as follows:

1. The intestinal microbiota is dynamic, self-stabilizing and resilient to the external addition of Clostridium cluster XIVa.
2. The effect of Clostridium cluster XIVa addition on microbiota is dose-dependent.
3. The repeated daily administration for 14 consecutive days is more efficient than a single dose of Clostridium cluster XIVa.
4. The changes in the microbiome are not persistent after a single dose or 14-day low dose treatment with Clostridium cluster XIVa; once the administration is stopped, a shift in the microbiome is no longer detected.
5. Laxative can create a favourable environment and enable a decrease in the required dose of Clostridium cluster XIVa to shift the type of the microbiome.
6. In the static status 5, Clostridium cluster XIVa can temporarily boost the abundance of Bacteroides if used in a single high dose or after laxative.

### Sequential laxative-probiotic usage is the best treatment for relief of IBS symptoms

**Figure 3:**
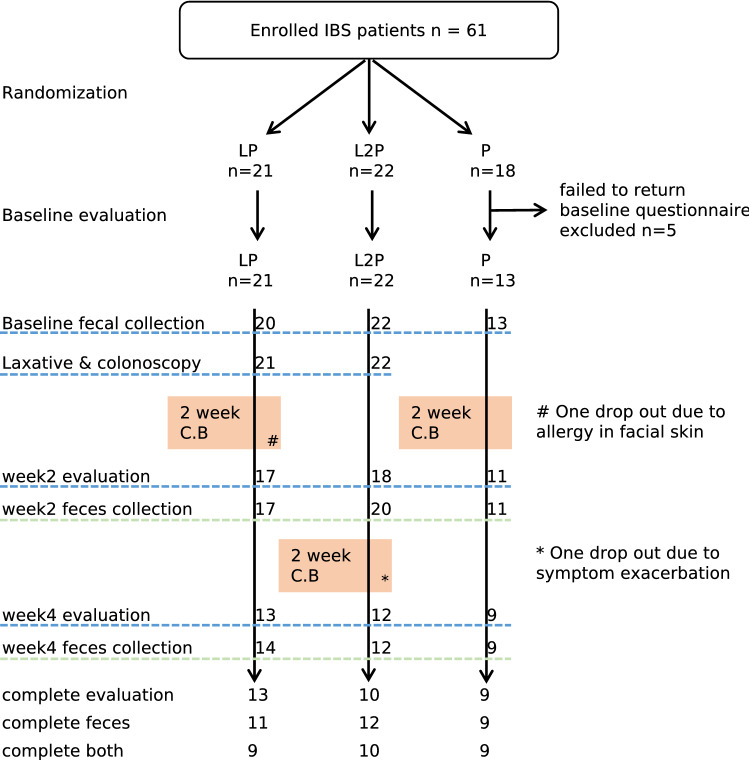
The flow chart and completeness of the exploratory trial.

Then, we determined whether coupled usage of laxative-probiotic can relieve the IBS symptoms better than conventional probiotic usage. A three-arm, randomized, open label preliminary clinical trial was carried out. The flowchart of the clinical trial is shown in Fig. 3. A total of 61 IBS patients were recruited, and after randomization and exclusion, 56 recipients were analysed in three groups; all patients received probiotic *C. butyricum* MIYA (C.B.) for 2 weeks in a variable type of administration: (1) LP group (n = 21): the patients received 2 L of laxative bowel preparation and were subjected to colonoscopy; the patients started the administration of probiotic C.B. for two weeks immediately after colonoscopy; (2) L2P group (n = 22): the patients received 2 L of laxative bowel preparation and were subjected to colonoscopy; the administration of probiotic C.B. started 2 weeks after the procedure; (3) P group (n = 13): the patients received no colonoscopy or laxative and started taking probiotic C.B. immediately after the enrolment. The patients were aware of the probiotic but were blinded to the aim of the study, which was to validate the mathematically predicted regimen. At baseline, there were no significant differences in age, gender, BMI, habits, disease subtype, individual or summed symptom score and disease history between the three groups (Table 1).

**Table 1:** Baseline demographics of enrolled patients.

Group		L2P	LP	P	P value
Sex (male/female)		13/9	11/10	7/6	0.9415
Mean age, years (range)		42.5 (18–69)	43.5 (18–64)	34.8 (24–55)	0.1192
Mean BMI (range)		22.2 (14.9–26.0)	24.3 (16.5–29.3)	23.0 (18.1–31.4)	0.0677
Number of patients		22	21	13	
IBS-D		21	19	13	0.6083
IBS-A		1	2	0	
Stool abnormality^c^		0.95 ± 0.08	0.90 ± 0.14	0.92 ± 0.18	0.9331
Abdominal pain	Severity^a^	2.64 ± 0.28	2.52 ± 0.36	1.62 ± 0.21	0.0615
	Frequency^b^	4.13 ± 0.54	2.71 ± 0.54	2.62 ± 0.64	0.1122
Abdominal discomfort	Severity	1.23 ± 0.23	2.14 ± 0.39	1.69 ± 0.24	0.2033
	Frequency	2.73 ± 0.62	2.71 ± 0.54	3.15 ± 0.55	0.5901
Bloating or distention	Severity	1.18 ± 0.27	2.05 ± 0.35	1.62 ± 0.31	0.1710
	Frequency	1.95 ± 0.56	2.33 ± 0.52	2.23 ± 0.64	0.3364
Urgency	Severity	2.45 ± 0.32	2.85 ± 0.38	2.15 ± 0.25	0.5288
	Frequency	3.77 ± 0.60	3.57 ± 0.56	3.92 ± 0.70	0.9051
Straining	Severity	0.55 ± 0.19	0.81 ± 0.32	1.15 ± 0.36	0.3142
	Frequency	0.91 ± 0.44	0.24 ± 0.10	1.23 ± 0.54	0.1348
Incomplete evacuation	Severity	1.27 ± 0.30	1.76 ± 0.36	1.77 ± 0.28	0.4762
	Frequency	3.45 ± 0.74	2.28 ± 0.58	3.15 ± 0.73	0.6191
Composite symptom score		9.32 ± 0.71	12.14 ± 1.34	10.00 ± 0.89	0.3433
Frequency-weighted symptom score		43.2 ± 5.8	41.7 ± 7.7	37.4 ± 6.4	0.3680
Impact on QOL		56.0 ± 3.3	59.9 ± 4.8	49.7 ± 2.1	0.4697
Disease history (years)		4.30 ± 0.99	5.48 ± 1.29	3.71 ± 1.02	0.7390
Self-reported association with stress		7/22	10/21	8/13	0.2186
Smoking		5/21	5/21	2/13	0.8134
Alcohol consumption		4/22	2/21	3/13	0.5452

^a^Shown as the mean score ± SEs.

^b^Shown as days per week.

Kruskal–Wallis test was used to compared continuous variable, and Fisher test was used to compared discrete variables.

^c^Stool abnormality was calculated as follows: 0 for Bristol score 3, 4, or 5; 1 for Bristol score 2 or 6; 2 for Bristol score 1 or 7.

**Figure 4:**
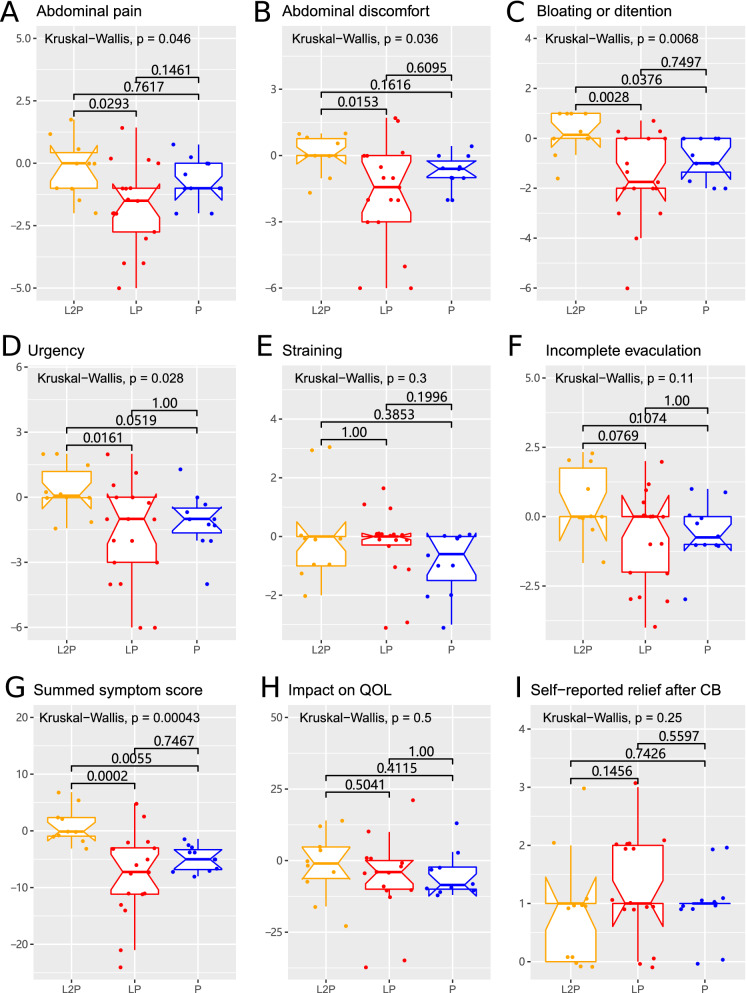
The symptoms, QOL, and self-reported relief during 2 weeks of *C. butyricum* intervention. A-F, the individual Likert-7 score changes during 2 weeks of *C. butyricum* intervention. The values correspond to the score at the end of week 2 minus the baseline score for the LP and P groups and to the score at the end of week 4 minus the score at the end of week 2 for the L2P group. Lower score corresponds to better condition of the patients. G and H, the changes in the summed symptom score and the impact on QOL during *C. butyricum* intervention for 2 weeks. Lower values correspond to the better score. F, the self-reported relief score at the end of *C. butyricum* intervention (week 2 for the LP and P groups and week 4 for the L2P group). Higher values correspond to better condition of the patients. The Kruskal–Wallis p value for overall comparison and the Dunn’s post hoc comparison p value are indicated in each figure. Dunn’s p < 0.025 indicates a significant difference between the two groups.

The changes in the symptom scores during two weeks of probiotic treatment or during the whole 4 weeks of the trial were compared. During two weeks of probiotic treatment, the patients in the LP group experienced the most pronounced relief in the severity of abdominal pain, abdominal discomfort, bloating or distention and urgency and in the summed symptom score (Fig. 4A–D and G, overall Kruskal–Wallis p < 0.05). Pairwise comparison of the three groups indicated that the LP group experienced more pronounced relief than that in the L2P group in terms of abdominal discomfort, bloating or distention, urgency and the summed symptom score (Fig. 4B–D and I, Bonferroni adjusted p < 0.025 according to Dunn’s post hoc test). Comparison of the data obtained at the end of week 4 to the baseline indicated that a reduction in the abdominal pain was more pronounced in the LP group compared to that in the P group but the difference was not significant (Fig S3A, overall Kruskal–Wallis (KW) p = 0.062, Bonferroni adjusted p < 0.0299). The median self-reported relief score was the highest in the LP group but the difference was not significant (Fig S3I, overall KW p = 0.18). These data suggest that C.B. has a better effect if the administration is started immediately after laxative treatment in IBS patients.

### Sequential laxative-C.B. usage optimally shifts the gut microbiota in IBS

**Figure 5:**
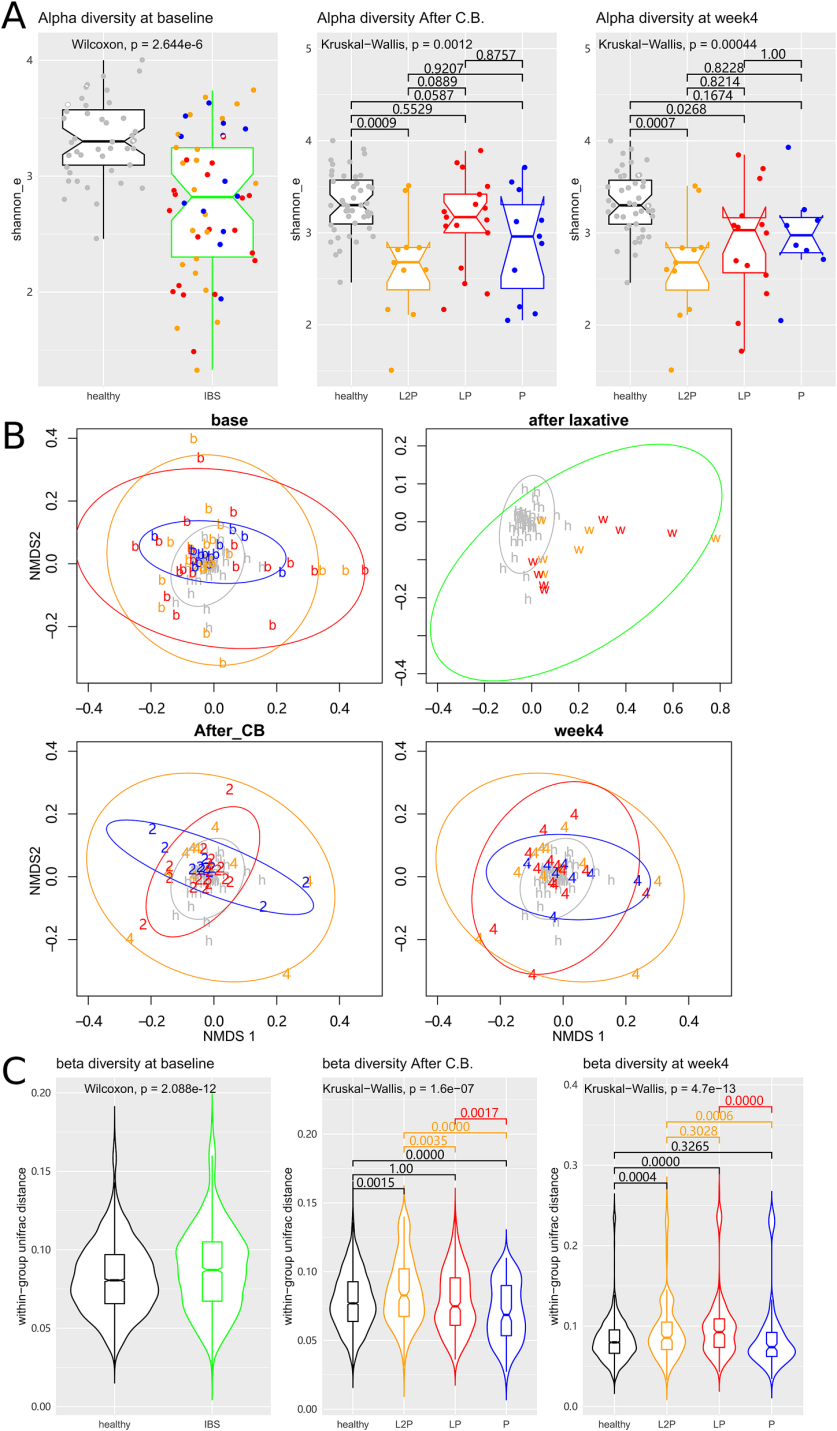
The microbiome response to the *C. butyricum* intervention. (**A**) The comparison of the alpha diversity composition. The p values of the Wilcoxon test or Kruskal–Wallis test with Dunn’s post hoc comparison are indicated in each plot. (**B**) The NMDS analysis is based on the Euclidean distance. (**C**) The comparison of the weighted UniFrac distances within the groups. The p values of the Wilcoxon test or Kruskal–Wallis test with Dunn’s post hoc comparison are indicated in each plot. Colours are consistent across the figure. Black colour and grey dots represent the healthy reference group. The L2P group is plotted in orange, the LP group is plotted in red, and the P group is plotted in blue. Green represents the mixed samples from the IBS recipients. The plot patch ‘b’ represents the baseline sample; ‘2’ corresponds to the end of week 2, ‘4’ corresponds to the end of week 4, ‘w’ represents the watery fecal samples after laxative, and ‘h’ corresponds to the healthy reference group.

Finally, we determined whether the faecal microbiota of IBS patients responds differently to the C.B. administration in the three groups. To evaluate the microbiome in the IBS cohort, the faecal 16S rRNA gene sequencing data were combined with that of a healthy reference group. This reference set was collected from 44 healthy individuals recruited in our centre and served as a common healthy reference microbiota dataset in our study (see Methods). Gender, age, and BMI of the healthy individuals were comparable to those in the IBS cohort (all p > 0.05, supplemental Table 1). Initially, the faecal microbiota diversity in the IBS cohort was compared to that in the healthy group. At baseline, the Shannon index of the IBS patients was lower than that of the healthy group (p = 2.644e-6, Wilcoxon test, Fig. 5a, left), which is consistent with previous reports. At the end of two weeks of C.B. administration, the sequential LP group, but not the L2P group, attained a higher alpha diversity level comparable to that of the healthy group (overall KW p = 0.0012, and Dunn’s post hoc comparison p = 0.5529 and 0.0009 for LP and L2P vs the healthy group, respectively, Fig. 5A, middle). At the end of week 4, the faecal diversity of the L2P group, but not of the LP or P groups, was lower than that of the healthy group (overall KW p = 0.00044, and Dunn’s post hoc comparison p = 0.0007 for L2P, 0.0268 for LP and 0.1674 for P vs healthy group, respectively, Fig. 5A, right). These data suggest that sequential usage of laxative and administration of C.B. for 2 weeks can boost the alpha diversity of the faecal microbiome in IBS to a normal-like level; however, 2-week interval between the use of a laxative and C.B. does not enhance restoration of the alpha diversity of the gut microbiota in IBS.

The community-level microbiome similarity in the IBS cohort was compared to that of the healthy group. Euclidean distance-based NMDS analysis of the operational taxonomic unit (OTU) relative abundance data was performed. The NMDS stress value was 0.1941. To describe the data in an intuitive manner, NMDS coordinates of each sample were plotted, and the ‘ordiellipse’ function of the vegan package of R was used to estimate the ellipse covering the 95% confidence region of the samples of each group in the NMDS plot (Fig. 5B). At the baseline, the faecal microbiota of the healthy group was relatively uniform and concentrated in a small region in the NMDS plot (grey ‘h’ and grey circle in Fig. 5B, upper left). In contrast, the baseline faecal samples of IBS patients were heterogeneous and distributed in a substantially larger ellipse (coloured ‘b’ and ellipses in Fig. 5B, upper left). This observation was confirmed by the comparison of the pairwise weighted UniFrac distances within each group (Fig. 5C, left). The baseline IBS samples had significantly larger UniFrac distances within the group than those in the healthy group (p = 2.088e-12, Wilcoxon test). The Euclidean distance-based Adonis test indicated that the baseline IBS microbiome was significantly different from that in the healthy group (P = 0.034, Table 2). These data suggest that the faecal IBS microbiome features a higher beta diversity compared to that in the healthy group.

**Table 2:** The Adonis test of the faecal microbiome of the patients.

Timeline	Comparison	Adonis F-value	df	Adonis P value
Baseline	IBS vs healthy	1.942	1	0.035*
Immediately after laxative	LP + L2P vs healthy	1.146	1	0.001*
After CB/week 4	L2P vs healthy	2.048	1	0.017*
After CB	LP vs healthy	1.203	1	0.266
After CB	P vs healthy	1.514	1	0.084
Week 4	LP vs healthy	1.306	1	0.143
Week 4	P vs healthy	1.398	1	0.109

Then, we evaluated whether the beta diversity of the IBS faecal microbiome responds differently to C.B. treatment in the three groups. In agreement with the previous reports, the laxative treatment significantly altered the individual faecal microbiomes (p = 0.001 Adonis test in Table 2; green ellipse vs grey ellipse in Fig. 5B, upper right). The LP group, which started C.B. treatment immediately after laxative administration, reached a less heterogeneous faecal microbiome resembling that of the healthy group at the end of 2 weeks of C.B. treatment according to the data of the NMDS plot (red vs grey ellipses, Fig. 5B, lower left). Consistently, the UniFrac distance within the group was approximately similar in the LP and healthy groups (overall KW p = 1.6e-7, Dunn’s post hoc test p = 1 for LP vs healthy, Fig. 5C, middle). The Adonis test showed that the microbial differences between the LP and healthy groups became insignificant after C.B. treatment (p = 0.266, Table 2). In contrast, the microbiome distribution was almost static in the L2P and P groups during the C.B. administration (Fig. 5B, orange and blue ellipses, respectively). At the end of week 4, the L2P group microbiome, but not the LP or P group microbiomes, remained significantly different with that of the healthy group according to Adonis test (Table 2). The beta diversity within the group was similar in the P and healthy groups at week 4, while the L2P and LP groups were more heterogeneous than the healthy group (overall KW p = 4.7e−13, Dunn’s post hoc comparison p = 0.0004 for L2P, p = 0.0000 for LP, and p = 0.3265 for P vs healthy groups, Fig. 5C, right). Overall, these data indicate that the start of oral C.B. administration may influence the individual responses of faecal microbiota, and starting C.B. treatment immediately after a laxative offers a unique opportunity to correct the IBS dysbiosis with a transient treatment.

Taxonomic composition of the faecal microbiota of IBS patients was compared to that of the healthy reference group, and in vivo responses were compared with the in silico simulation. The major phyla and genera at the baseline are compared in Table 3, and the full list is presented in the Supplemental data. The abundance of the Proteobacteria phylum was lower in IBS patients than that in the healthy group (median 2.88 vs 8.01, FDR-adjusted p = 0.040). Genus level comparison did not reveal significant differences except Bifidobacterium that is overrepresented in IBS (FDR-adjusted p = 0.038). The content of Bacteroides tends to be lower in IBS patients (unadjusted p = 0.036) and can be transiently boosted in silico. However, the results of the in vivo trial did not demonstrate a significant increase in the content of Bacteroides (Table 3).

**Table 3:** The taxonomic composition of the faecal microbiome of the IBS group compared to that of the healthy group.

	χ^2^	p (KW)	P adjusted (FDR)	Healthy (median and interquartile range)	IBS baseline (median and interquartile range )
Phylum %					
Firmicutes	1.156	0.282	0.392	73.9 (50.75–83.18)	78.25 (58.28–87.55)
Bacteroidetes	2.113	0.146	0.280	10.85 (3.39–23.88)	5.39 (2.32–13.78)
Proteobacteria	7.022	0.008	0.040	8.01 (1.56–26.83)	2.88 (0.9–7.78)
Actinobacteria	20.572	0.000	0.000	0.51 (0.22–1.64)	2.84 (1.16–6.22)
Genus %					
Faecalibacterium	5.290	0.021	0.316	4.64 (0.57–10.63)	9.71 (3.25–17.88)
Blautia	0.314	0.575	0.811	8.87 (1.66–16.85)	7.04 (2.88–10.8)
Escherichia/Shigella	5.356	0.021	0.316	1.78 (0.57–17.18)	0.82 (0.26–4.47)
Bacteroides	4.410	0.036	0.445	5.25 (2.28–14.63)	3.19 (0.52–6.42)
Prevotella	1.623	0.203	0.668	0 (0–0.72)	0.02 (0–0.55)
Bifidobacterium	13.514	0.000	0.038	0.35 (0.04–1.13)	2.25 (0.23–5.56)
Gemmiger	5.891	0.015	0.316	0.84 (0.03–2.39)	2.39 (0.46–7.27)

	χ^2^	p	Healthy vs	Abundance	
Genus %					
Bacteroides	2.105	0.150 (KW)	IBS combined after C.B	2.93 (0.67–8.85)	
	3.045 (KW p = 0.38)	1.000 (Dunn, Bonferroni)	L2P after C.B	5.360 (1.335–12.00)	
		0.6324 (Dunn, Bonferroni)	LP after C.B	2.930 (0.560–4.860)	
		0.4351 (Dunn, Bonferroni)	P after C.B	1.960 (0.695–6.290)	

## Discussion

In this study, an NEDN model was evolved from the classical gLV model and is proposed to describe the dynamic changes based on the previously published faecal microbial data. The NEDN model was used to simulate the stability of the gut microbiome and to determine an optimal probiotic intervention approach. The unbalanced low-mass gut microbiota created by the laxative bowel cleaning greatly decreased the intensity threshold of probiotic administration required to shift the gut microbiome. A small-scale preliminary clinical trial was conducted, and the results validate the hypothesis that the sequential laxative-probiotic usage produces an optimal decrease in the IBS symptom score during 2-week C.B. administration. The individual gut microbiome responses to the C.B. treatment depend on the starting status with regard to the laxative bowel cleaning, and the sequential LP group attained a temporal microbial status at the end of the 2-week C.B. administration that closely resembles the healthy reference group microbiome.

This study emphasizes that the time of the initiation of probiotic administration may influence treatment efficacy, which was overlooked in the majority of previous clinical trials that assessed the benefits of probiotics. Given the complexity of the gut microbiota, its response to external intervention is likely to be dependent on the composition of microbiota. Bennet et al. reported that faecal bacterial profiles of patients with IBS predicted their responsiveness to a diet low in fermentable oligosaccharides, disaccharides, monosaccharides and polyols (FODMAPs)^16^. Another previous study showed that the effect of a low FODMAP diet was dependent on the faecal volatile organic compounds in the recipients^17^. In agreement with these reports, our mathematical simulation and clinical trial data suggest that the laxative bowel purge facilitates the probiotic effect on the gut microbiota. The laxative bowel cleaning may serve as a preparation procedure prior to colonoscopy in IBS patients and may be a part of the treatment targeting the gut microbiome. The preliminary data of this study suggest that if the initiation of probiotic treatment is consciously controlled, the patients gain additional benefit from the probiotics without increasing their cost. In our previous meta-analysis^18^, probiotic therapy was associated with more pronounced improvement than that achieved by placebo administration in terms of the overall symptomatic response but not in terms of the individual IBS symptoms. In this study, abdominal pain, abdominal discomfort, bloating/distention and defecation urgency symptoms and the summed symptom score were optimally relieved in the LP group. Although the cohort is small, the additional benefits of the sequential laxative-probiotic usage warrant a follow-up trial in IBS patients.

In this study, C.B. was used as a single probiotic strain. Our previous study^19^ documented that *C. butyricum* improves overall symptoms, quality of life and stool frequency in IBS-D patients. Meanwhile, the responder rate for C.B. was only 44.76%^19^. Thus, it may be possible to optimize the usage of *C. butyricum* to potentially increase its efficiency. On the other hand, MIYA is a single-strain probiotic, and this type of treatment is easier to initiate in a modelling study. In the simulation section, Clostridium cluster XIVa was selected as the “probiotic treatment” for the following reasons. 1) Some species of Clostridium cluster XIVa are butyrate producers^20^ functionally similar to C.B. 2) The 16s rRNA gene of C.B. is assigned to the “p:Firmicutes, c:Clostridia, o:Clostridiales, f:Clostridiaceae_1, g:Clostridium_sensu_stricto” taxonomy, which is similar to Clostridium cluster XIVa used in the NEDN model. Whether an effect of sequential laxative-C.B. usage applies to other probiotics and their efficacy in a large IBS cohort should be investigated in a future study.

This study emphasizes the importance of multidisciplinary cooperation for precise management of the gut microbiota. The optimal strain combination, dose and duration of probiotic usage in IBS patients are unclear. However, the gut microbiome consists of approximately one thousand species, and there are more than 20 commercially available probiotic strains. Possible combination of these parameters is an overwhelmingly vast array that is not feasible to assess by physicians in clinical trials. Here, we demonstrated that mathematical models may guide the search for an optimal probiotic regimen. On the other hand, mathematical models can only infer the changes in the microbial composition but cannot predict the changes in the symptoms in patients because the latter involves substantially more complex mechanisms, including barrier, immune, neural and psychological factors. Thus, the assumptions produced by mathematical models need to be thoroughly tested in clinical trials. Close cooperation between physicians and basic science researchers may help to solve this problem. This study has two important limitations. During this study, the performance of the NEDN model was not perfect and not suitable for simulation of complex intervention combinations. However, clinicians were unable to provide sufficient data to generate models containing higher number of genera. The parameters for the laxative bowel cleaning were set arbitrarily rather than by actually measuring the remaining biomass in the gut. This limitation delayed the progress of this study for several years. Refined gut microbiome models in combination with larger clinical trials may provide new insight in gut microbiome management in the future.

Several other limitations of this study should be considered. Most importantly, the phase II study was an open label clinical trial without a placebo control. Unlike strictly controlled and blinded clinical trials, the design of this study aimed to compare the initiation times of C.B. intervention. Thus, this study provides only incremental knowledge on probiotic management in IBS. Additionally, the recipients of the phase II study were recruited in a single centre and at a small scale, and the 4-week follow-up duration was relatively short. These two issues might have cause a potential bias. Hence, this study should be regarded only as an exploratory investigation. Despite these shortcomings, our results demonstrate previously unknown features of microbial dynamics and probiotic usage. The novel findings of this study warrant further validation, especially in large clinical trials.

In conclusion, the combination of laxative bowel cleaning and sequential oral *C. butyricum* intake optimally reduced the symptoms in IBS patients and transiently corrected their dysbiosis. Translation and application of mathematical models may lead to precision medicine targeting of the gut microbiome.

## Materials and methods

### Time-series data and mathematical modelling

The publicly available dense time-series microbiome data of Caporaso et al. were downloaded from MG-RAST (4457768.3–4459735.3)^15^. This dataset includes the 16s rRNA gene sequencing data and the corresponding metadata of 1967 microbial samples collected from 2 individuals (M3 and F4). To enhance the homogeneity of microbial dynamics, only 336 faecal microbiome data entries from a single recipient (M3) were included. This was the longest and most dense dataset of time series of the human gut faecal microbiome available at the time of the initiation of this study. Four potentially mislabelled samples were excluded. The remaining 332 faecal samples were collected from 2008-10-21 to 2010-01-06. The raw sequencing data of the remaining 332 samples were processed, clustered, and analysed similar to our own sequencing data (see sequencing data processing section). These data generated 1,270 OTUs assigned to 119 genera. Average abundance of each genus across the dataset was calculated. The 12 genera with an average abundance above 1% and an “other” genus representing the rest of the microbiome were used to build the mathematical model.

The next-day faecal microbiome data were available in 269 out of 332 faecal samples. Thus, 269 day0–day1 data pairs were defined to train the microbiome model. The NEDN model had 169 parameters, including 13 inherent growth rates for vector α and 156 parameters of a 13*13 zero-diagonal matrix β. All parameters were constrained within [−3, 3]. The parameters were evaluated by a function summing the squared differences between the predicted next-day abundance of a genus and the actual abundance. The parameters were optimized using the NLopt v2.61 and the nloptr tool in R. The “NLOPT_GN_CRS2_LM” algorithm was selected as the optimization method^21^, and the termination conditions were set as "xtol_rel" = 1.0e-12 and "ftol_rel" = 1.0e-12. A total of 57,705,618 iterations were required to obtain the optimal parameters.

To compare the stability of the NEDN model with that of the classical gLV model, the gLV model was fit to the same dataset. The gLV model included 13 inherent growth rates as vector α and 156 inter-genus interactions in the form of the 13*13 zero-diagonal matrix β; all parameters were constrained within [− 3, 3] similar to the NEDN model. In contrast to the NEDN model, the gLV model did not limit abundance of a genus to positive values and did not force the sum of genus abundance to be 1. The equation of the gLV model was:

$$\overrightarrow{{x}_{t+1}}=\overrightarrow{{x}_{t}}\times [1+\overrightarrow{\alpha }\cdot (1-\overrightarrow{{x}_{t}})+\overrightarrow{{x}_{t}}\times \beta ]$$

where the vector *x*_t_ represents the relative abundance of modelled genera in a current faecal sample; α and β are the inherent growth rate and interaction matrix, respectively. Both models were fit to the next-day data pairs, and the accumulated prediction accuracy was evaluated based on the day0–day2 and day0–day3 data pairs. A total of 266 day0–day2 and 266 day0–day3 microbiome pairs were extracted from the published dataset^15^*.* The day 2 or day 3 microbiomes were predicted using the NEDN or gLV models, and the results were compared with the actual measurement.

### In silico simulation and the static microbiome search

The dynamic changes in the microbiome were simulated by calculating the day-to-next-day changes using the NEDN model iteratively. To exhaustively search for the possible *static* composition of the faecal microbiome, 1000 random microbiomes were generated in the form of 13-dimensional Dirichlet distribution with shape parameter vector 0.2 for each dimension (using the rdirichlet function in the MCMCpack package of R). This random microbiome was combined with 332 actual microbiomes in the previous dataset^15^ as the starting microbiome. Dynamic changes in this starting microbiome were simulated for 400 days. If the microbiome remained almost unchanged in the last 100 days (the sum of squares of the variance for each genus are less than 10^–20^), the microbiome was considered static. The NMDS and cluster analysis of the random and actual microbiome was performed in R.

The microbiome responses to two types of hypothetical interventions were simulated. (1) Oral administration of probiotic belonging to the genus Clostridium cluster XIVa was simulated by adding a given percentage to the abundance of Clostridium cluster XIVa and then normalizing the total microbial abundance to 1. The administration was simulated once on day 50 or 51 or on a daily basis starting from day 51 to day 64. (2) Laxative treatment was simulated by multiplying all values of genus abundance by 1% on day 50. This treatment was simulated alone or was followed by sequential probiotic administration.

### Clinical trial design

IBS patients were included in the three-arm, open label, randomized clinical trial to explore whether coupled laxative-probiotic usage can efficiently relieve IBS symptoms. According to Excel-generated random numbers, the recipients were randomized to three groups: (1) LP group: the patients received 2 L of laxative bowel preparation and were subjected to colonoscopy; the patients started the administration of probiotic C.B. for two weeks immediately after colonoscopy; (2) L2P group: the patients received 2 L of laxative bowel preparation and were subjected to colonoscopy; the administration of probiotic C.B. started 2 weeks after the procedure; (3) P group: the patients received no colonoscopy or laxative and started taking probiotic C.B. immediately after the enrolment. Patients took 2 tablets of C.B. 3 times daily for 2 weeks. No other medications, including prebiotics or symptom-relieving drugs, were given during their participation in the trial for 4 weeks. The patients were instructed to maintain their usual eating habits and avoid drastic diet changes during the trial. The patients were aware of the probiotics they took, but they were blinded to the hypothesis of this study. All patients were examined at baseline and at 2 and 4 weeks to complete the IBS Likert-7 symptom score system and IBS-specific quality of life (IBS-QoL) questionnaires^22,23^. Additionally, the patients were asked to keep a daily diary including symptoms, frequency of defecation, Bristol stool form scale recording and any side effect of the treatment. The primary endpoint was to evaluate the global assessment of the relief by the patients during probiotic usage for 2 weeks and the overall assessment after 4 weeks. The secondary endpoint was to evaluate the changes in the symptom scores, quality of life of IBS patients, and composition of microorganisms in the stool during the usage of probiotics for 2 and 4 weeks.

Adult patients with IBS (aged 18–70 years) were recruited from July 2014 to February 2015 at the Department of Gastroenterology, Shandong University Qilu Hospital. Patients were diagnosed with IBS according to the Rome III criteria. The inclusion criteria were as follows:

- Patients identified by Rome III criteria for IBS.
- Patients scheduled for colonoscopy or negative screening examinations.
- Patients from 18 to 70 years of age.

The exclusion criteria were as follows:

- Antibiotic, probiotic or laxative usage within 4 weeks.
- Organic gastrointestinal diseases.
- Severe systematic disease: diabetes mellitus, hepatic, renal or cardiac dysfunction, thyroid disease or tumour, etc.
- Pregnancy or lactation.
- Previous major or complicated abdominal surgery.
- Severe endometriosis and dementia.

The patients were classified into three IBS subtypes: constipation-predominant (IBS-C), diarrhoea-predominant or alternating periods of constipation and diarrhoea (IBS-M)^24^.

This study was compliant with the requirements of good clinical practice and the revised Declaration of Helsinki. The ethics committee of Qilu Hospital approved this trial (approval number: 201430). The study was registered at www.clinicaltrials.gov (NCT02254629). All participants provided a written informed consent to participate after obtaining verbal and written information about the study. Participants were able to discontinue their participation in the trial at any time point on their request. Because of the preliminary nature of this study and limited 16S sequencing budget, we terminated the recruitment of the recipients when 60 subjects were enrolled.

### Faecal sample collection and sequencing

Faecal samples were longitudinally collected from the patients at baseline before bowel cleaning and at the end of weeks 2 and 4. The collected samples were immediately frozen at -80 ℃ and transferred to Majorbio (Shanghai, China), where the total DNA was extracted and treated according to the standardized protocol for subsequent analysis^25^. The V3- > 4 region of the 16S ribosomal subunit gene was amplified using the paired 338F and 806R barcoded primers and sequenced using an Illumina PE300 platform. The raw reads were filtered using the following criteria. (1) The low-quality bases in the tail of raw reads were cut off. If the average base quality in a sliding window of 50 bp was lower than 20, the bases after the beginning of the window were cut off. If the remaining read was shorter than 50 bp, the whole read was discarded. (2) The paired reads were merged if the minimal overlap length was 10 bp. (3) The maximum allowed mismatch ratio in the overlap region was 0.2. 4) The merged reads were assigned to the samples according to the barcodes at both ends. The maximum allowed mismatch of the barcodes was 0, and the maximum allowed mismatch of the primers was 2.

### Sequence processing and microbiomics analysis

To evaluate the faecal microbiome of the IBS recipients, the sequencing data were merged with those of the healthy reference group. Healthy group included 44 faecal microbiomes from healthy recipients obtained in our previous study (PRJNA544721). All valid sequencing data were prepared using usearch v10.0.240 according to the pipeline example (https://drive5.com/usearch/manual/ex_miseq.html )^26^. Briefly, the valid sequences were dereplicated by the fastx_uniques algorithm of usearch. The cluster_otus algorithm of usearch was used to cluster the dereplicated sequences to the same OTU if the distance was less than 0.03. Then, a representative sequence of each OTU was aligned using RDP (release 11.5) by the sintax algorithm with the sintax_cutoff parameter of 0.8. The OTU abundance values were merged at the genus and phylum levels using the sintax_summary algorithm with parameters -rank g and -rank p, respectively. The sequencing results for this IBS cohort were archived in the Short Reads Archive (PRJNA573815). The alpha and beta diversity were calculated using the usearch -alpha_div, -cluster_agg, and -beta_div algorithms.

### Statistical methods

All statistical analysis was performed in RStudio (v1.1.463), the integrated development environment for R (v.3.51). For clustering analysis, the Bray distance of the microbiome was calculated using the vegdist function of the vegan package, and the hierarchical cluster analysis was performed using the "ward.D" algorithms of the hclust function. The comparison of the continuous values between multiple groups was performed by the dunn.test algorithm with parameter [method = "bonferroni", altp = FALSE], which includes the Kruskal–Wallis test and Dunn’s post hoc comparison with Bonferroni correction of p values. In Dunn’s post hoc comparison, the null hypothesis (H0) was rejected only when the p values were less than α/2 (0.025). Wilcoxon test was used for comparison of continuous values between two groups. Categorical values, such as sex and IBS subtype, we compared using the fisher.test algorithm in R. The OTU abundance, taxonomy profile, diversity profile and metadata were evaluated in RStudio. Kruskal–Wallis test was used to compare the genus and phylum level abundance of the microbiomes in the IBS and healthy sets. The resulting p values were adjusted using the FDR method of the p.adjust function in R.

## Supplementary information

Supplementary Information 1.

Supplementary Information 2.

Supplementary Information 3.

Supplementary Information 4.

Supplementary Information 5.

## Data Availability

The sequencing data were archived in the Short Reads Archive. The reads of the IBS recipient faecal samples were deposited under PRJNA573815. The reads of the healthy reference faecal samples were deposited under PRJNA544721.
