# Supplementary material for: Sequential laxative-probiotic usage for treatment of irritable bowel syndrome: a novel method inspired by mathematical modelling of the microbiome
Source: Sci Rep. 2020 Nov 9;10:19291. doi: 10.1038/s41598-020-75225-z (PMC7652883; doi:10.1038/s41598-020-75225-z)
Supplement: Supplementary file 1 — Supplementary Information 1. [file 41598_2020_75225_MOESM1_ESM.docx]

Sequential laxative-probiotic usage for treatment of irritable bowel syndrome: A novel method inspired by mathematical modelling of the microbiome

**Authors:** Ming Li^1†^, Ri Xu^1†^, Yan-qing Li^1*^

† These authors contributed equally to this work.

**Affiliations:**

^1^ Department of Gastroenterology, Qilu Hospital of Shandong University, Jinan, 250012, China.

*To whom correspondence should be addressed: Yan-qing Li

Department of Gastroenterology, Qilu Hospital of Shandong University

107 Wenhuaxi Road, Jinan, China

250012

Fax: +86-531-82166090

Email: liyanqing@sdu.edu.cn.

Ruminococcus

A

Roseburia Prevotella

Phascolarctobacterium Parabacteroides

other Faecalibacterium Clostridium_XlVa

Blautia Bacteroides Alistipes Akkermansia (Unassigned)

−0.3−0.2−0.1 0.0

# inherent growth rate

other Clostridium_XlVa

B

Blautia

# Interaction

Phascolarctobacterium −3

Faecalibacterium −2

Akkermansia −1

Ruminococcus

Prevotella 0

Alistipes 1

Roseburia 2

Parabacteroides

(Unassigned) 3

Bacteroides

**Fig. S1.** The parameters of the gLV model. The inherent growth rate (vector α, A) and the inter-genus interaction (matrix β, B) for the genera in the gLV model.
