# Supplementary material for: Sequential laxative-probiotic usage for treatment of irritable bowel syndrome: a novel method inspired by mathematical modelling of the microbiome
Source: Sci Rep. 2020 Nov 9;10:19291. doi: 10.1038/s41598-020-75225-z (PMC7652883; doi:10.1038/s41598-020-75225-z)
Supplement: Supplementary file 2 — Supplementary Information 2. [file 41598_2020_75225_MOESM2_ESM.docx]

Sequential laxative-probiotic usage for treatment of irritable bowel syndrome: A novel method inspired by mathematical modelling of the microbiome

**Authors:** Ming Li^1†^, Ri Xu^1†^, Yan-qing Li^1*^

† These authors contributed equally to this work.

**Affiliations:**

^1^ Department of Gastroenterology, Qilu Hospital of Shandong University, Jinan, 250012, China.

*To whom correspondence should be addressed: Yan-qing Li

Department of Gastroenterology, Qilu Hospital of Shandong University

107 Wenhuaxi Road, Jinan, China

250012

Fax: +86-531-82166090

Email: liyanqing@sdu.edu.cn.

Bacteroides day+3 error

C


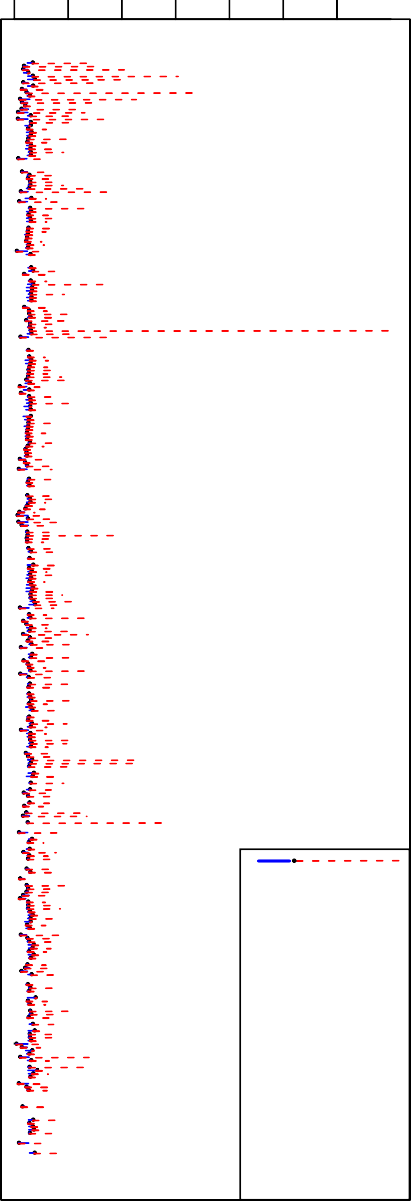
0 2 4 6 8 10 14

Bacteroides day+2 error

0 1 2 3

B

Bacteroides day+1 error

0.2 0.6 1.0

A

abundance predicted by gLV

actual measurement abundance predicted by NEDN


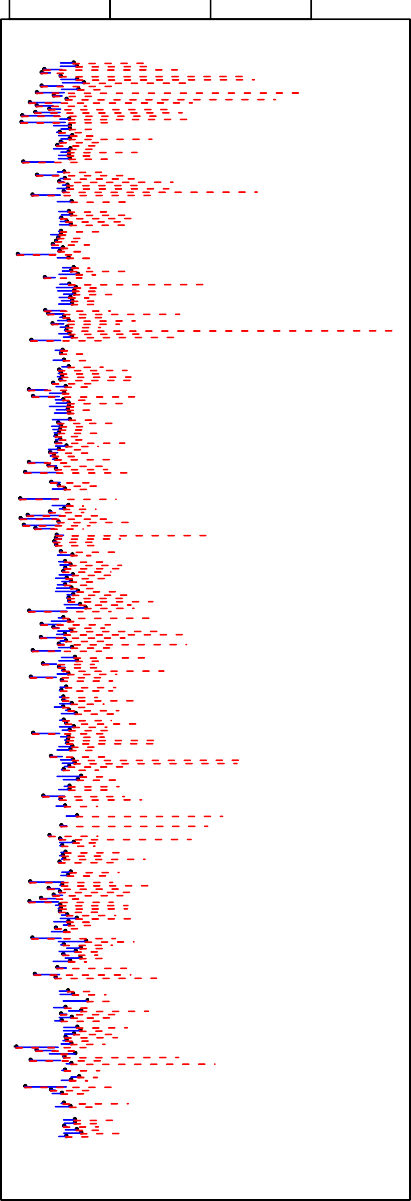

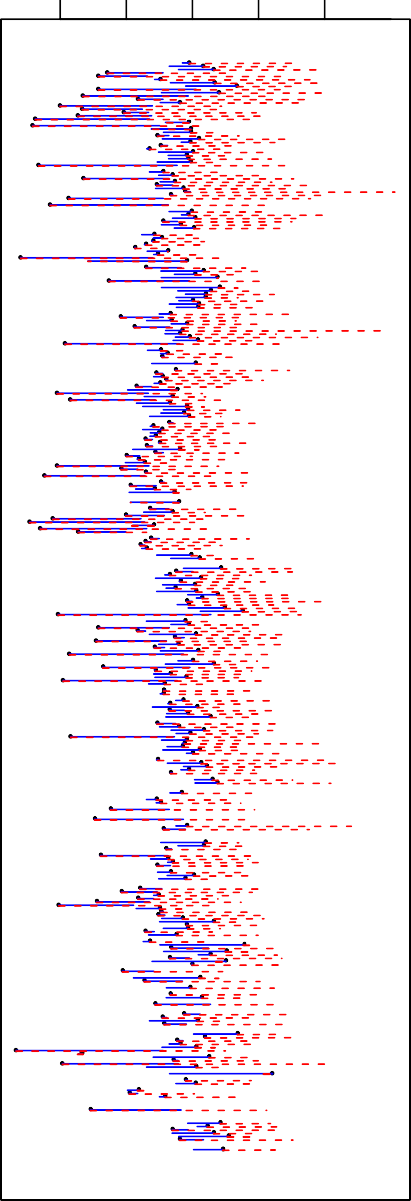


**Fig. S2.** The prediction error of the NEDN model and the gLV model. The abundance of Bacteroides was predicted by NEDN or gLV model from 1 day before (A), 2 days before (B), and 3 days before (C). The black dot represents the actual abundance of Bacteroides. The blue solid line represents the prediction error by the NEDN model. The red dashed line represents the prediction error by the gLV model.
