# Supplementary material for: Sequential laxative-probiotic usage for treatment of irritable bowel syndrome: a novel method inspired by mathematical modelling of the microbiome
Source: Sci Rep. 2020 Nov 9;10:19291. doi: 10.1038/s41598-020-75225-z (PMC7652883; doi:10.1038/s41598-020-75225-z)
Supplement: Supplementary file 3 — Supplementary Information 3. [file 41598_2020_75225_MOESM3_ESM.docx]

Sequential laxative-probiotic usage for treatment of irritable bowel syndrome: A novel method inspired by mathematical modelling of the microbiome

**Authors:** Ming Li^1†^, Ri Xu^1†^, Yan-qing Li^1*^

† These authors contributed equally to this work.

**Affiliations:**

^1^ Department of Gastroenterology, Qilu Hospital of Shandong University, Jinan, 250012, China.

*To whom correspondence should be addressed: Yan-qing Li

Department of Gastroenterology, Qilu Hospital of Shandong University

107 Wenhuaxi Road, Jinan, China

250012

Fax: +86-531-82166090

Email: liyanqing@sdu.edu.cn.

A Abdominal pain

●

● ●

●

●

●

● ● ●

●

●

● ●

●

●

●

**●**●

●

●

● ●

●

●

●

●

●

●

●

●

●

Kruskal−Wallis, p = 0.062

0.0299

0.1488

0.7211

●

4

B Abdominal discomfort

6

●

●

●

**●** ● ● ●

● ●

●

●

●●

●

●

●

●

● ●

●

●

●

●**●** ●

●

●

●

**●**

Kruskal−Wallis, p = 0.41

0.5974

1.00

0.2871

●

●

3

C Bloating or distention

2

Kruskal−Wallis, p = 0.17

0.6376

5

●

● ●

●

●

●

●

●

●

● ●●

●●

●

●

●

● ●●

●

●● ● ●

●

●

●

●

● ●

●

●

0.0901

343

0.

0 0 0

−3

−4 −2

5.0

D

2.5

0.0

−2.5

−5.0

L2P LP P

Urgency

●

●

●

● ●

●

●

●

● ●

●

● ●

●

●

●

● ●

●

●

●

●

●●

● ● ● ●

●

●

●

●

Kruskal−Wallis, p = 0.75

0.9065

1.00

0.6955

●

−6

L2P LP P

E Straining

| Krus | kal−Wallis, | p = 0.13 | |  |
| --- | --- | --- | --- | --- |
|  | 0.0 | 0.3377 | |  |
|  |  | 669 | |  |
|  | 0.5533 |  | |  |
|  |  |  |  |  |
|  | **●** |  | |  |
| ● ● | **●** | ● | | ● |
| ● **●** ● | ● **●** ●  **●** | ● ● | ● | ● |
|  |  |  | | ● ● |
|  | ● |  |  | ● |
|  | ● |  |  | ● |
| ● |  | ● | |  |
|  |  |  | | ● |
|  |  | ● | |  |

4

2

0

−2

# F

5.0

2.5

0.0

−2.5

L2P LP P

Incomplete evaculation

Kruskal−Wallis, p = 0.67

1.00

9

●

●

●

●

●

●

● ● ●

●

●

● ●●

●

●

●

●

●

●

●

● ● ●

●

●

**●** ●

●

●

●

0.5682

043

0.

# G

20

10

0

−10

−20

L2P LP P

Summed symptom score

●

●

●

●

●

●

● ●

●

●

●

● ● ●●

●

● ●

●

● ●

●

●

●

●

●

● ●

●

●

●

Kruskal−Wallis, p = 0.46

0.5464

1.00

0.3582

●

●

●

L2P LP P

L2P LP P

H Impact on QOL

●

● ●

●

●

●

● ●●

●

●

●

●

●

●

● ●

●

●

●

●

● ●

● ●

●

●

● ●

●

Kruskal−Wallis, p = 0.23

0.8308

0.4891

0.1326

●

●

50

0

L2P LP P

L2P LP P

I Self−reported relief at week4

5

| Krus | kal−Wallis, | p = 0.18 |  |
| --- | --- | --- | --- |
|  | 0. | 0.1028 |  |
|  |  | 383 |  |
|  | 0.4307 |  |  |
|  |  |  |  |
| ● |  |  |  |
| ● | ●● ● | ● ● |  |
|  |  | ● ● | ● |
| ●● ● ● | ● | ● |  |
|  |  |  |  |
| ● | ● ● | ●  ● ● | ● ● |
| ● ● | ● | ● | **●** |

4

3

2

1

0

L2P LP P

**Fig. S3.** The symptom, QOL, and the self-reported relief during the 4-week observation. A-F, The individual symptom score changes during the 4-week trial. G, The summed symptom score change during the 4 weeks. H, The change of the impact on QOL during the 4 weeks. I, The self−reported relief at the end of week 4.
