# Supplementary material for: Sequential laxative-probiotic usage for treatment of irritable bowel syndrome: a novel method inspired by mathematical modelling of the microbiome
Source: Sci Rep. 2020 Nov 9;10:19291. doi: 10.1038/s41598-020-75225-z (PMC7652883; doi:10.1038/s41598-020-75225-z)
Supplement: Supplementary file 4 — Supplementary Information 4. [file 41598_2020_75225_MOESM4_ESM.docx]

Sequential laxative-probiotic usage for treatment of irritable bowel syndrome: A novel method inspired by mathematical modelling of the microbiome

**Authors:** Ming Li^1†^, Ri Xu^1†^, Yan-qing Li^1*^

† These authors contributed equally to this work.

**Affiliations:**

^1^ Department of Gastroenterology, Qilu Hospital of Shandong University, Jinan, 250012, China.

*To whom correspondence should be addressed: Yan-qing Li

Department of Gastroenterology, Qilu Hospital of Shandong University

107 Wenhuaxi Road, Jinan, China

250012

Fax: +86-531-82166090

Email: liyanqing@sdu.edu.cn.

Table S1. The demographic comparison between the IBS cohort and the healthy control set.

|  | IBS cohort | healthy control | p-value |
| --- | --- | --- | --- |
| Sex (male/female) | 31/25 | 25/19 | 1 (Fisher) |
| Median age, year (range) | 42.5 (18-69) | 34.5 (22-65) | 0.428 (Wilcoxon) |
| Median BMI, (range) | 23.6 (14.9-31.4) | 23.2 (18.1-34.0) | 0.731 (Wilcoxon) |
