# Supplementary material for: Sequential laxative-probiotic usage for treatment of irritable bowel syndrome: a novel method inspired by mathematical modelling of the microbiome
Source: Sci Rep. 2020 Nov 9;10:19291. doi: 10.1038/s41598-020-75225-z (PMC7652883; doi:10.1038/s41598-020-75225-z)
Supplement: Supplementary file 5 — Supplementary Information 5. [file 41598_2020_75225_MOESM5_ESM.pdf]

Sequential laxative-probiotic usage for treatment of irritable bowel syndrome: A novel method inspired by mathematical modelling of the microbiome

Authors: Ming Li1†, Ri Xu1†, Yan-qing Li1*
† These authors contributed equally to this work.

Affiliations:
1 Department of Gastroenterology, Qilu Hospital of Shandong University, Jinan, 250012, China.
*To whom correspondence should be addressed: Yan-qing Li
Department of Gastroenterology, Qilu Hospital of Shandong University
107 Wenhuaxi Road, Jinan, China
250012
Fax: +86-531-82166090
Email: liyanqing@sdu.edu.cn.

phylum	chi_squared	p (KW)	p.adjust (FDR	healthy.media	healthy.1st_Q	healthy.3rd_Q	ibs.median	ibs.1st_Qu.	ibs.3rd_Qu.
Firmicutes	1.15552919	0.28239468	0.39225752	73.9	50.75	83.175	78.25	58.275	87.55
Bacteroidetes	2.1127082	0.14608059	0.27992707	10.85	3.39	23.875	5.385	2.3175	13.775
Proteobacteria	7.02214165	0.00805079	0.04025394	8.01	1.555	26.825	2.875	0.9	7.775
Actinobacteria	20.5724413	5.74E-06	5.74E-05	0.51	0.215	1.6425	2.835	1.16	6.2175
(Unassigned)	1.01458878	0.31380601	0.39225752	0	0	0.0325	0	0	0.145
Verrucomicrobia	0.25644469	0.61257329	0.68063699	0	0	0	0	0	0.0075
Fusobacteria	1.9010911	0.16795624	0.27992707	0	0	0.01	0	0	0
Candidatus_Saccharibacteria	4.07265113	0.04358282	0.14527607	0.01	0	0.0325	0.03	0.01	0.06
Cyanobacteria/Chloroplast	2.50024065	0.1138289	0.27992707	0	0	0	0	0	0
Synergistetes	0.16658991	0.68316042	0.68316042	0	0	0	0	0	0
Euryarchaeota	NA	NA	NA	0	0	0	0	0	0
Chloroflexi	NA	NA	NA	0	0	0	0	0	0
Deinococcus-Thermus	NA	NA	NA	0	0	0	0	0	0

genus	chi_squared	p (KW)	p.adjust (FDR)
(Unassigned)	0.268162336	0.604567165	0.826973943
Faecalibacterium	5.289966157	0.021448637	0.315879925
Blautia	0.314382462	0.5750033	0.810621117
Escherichia/Shigella	5.356293934	0.020647607	0.315879925
Bacteroides	4.409746848	0.035734144	0.445302406
Prevotella	1.622763403	0.202706901	0.667784148
Bifidobacterium	13.51415545	0.000236771	0.038356848
Gemmiger	5.891330715	0.015215598	0.315879925
Romboutsia	0.011480763	0.914671313	0.974847057
Megamonas	0.006347619	0.936498171	0.977056008
Ruminococcus	2.56853192	0.109008854	0.667784148
Lachnospiracea_incert	8.894763846	0.002859896	0.115825784
Fusicatenibacter	0.418296632	0.517788218	0.765473699
Roseburia	0.05561335	0.813568601	0.940668859
Phascolarctobacterium	1.391705118	0.238117357	0.667784148
Dialister	1.147587201	0.284054803	0.672793891
Bacillus	1.451074796	0.228355571	0.667784148
Dorea	1.364917231	0.242687097	0.667784148
Anaerostipes	1.671746041	0.19602483	0.667784148
Clostridium_sensu_stri	0.163169531	0.686255014	0.848651239
Streptococcus	2.094158486	0.147863105	0.667784148
Ruminococcus2	0.607311927	0.435801716	0.697030266
Clostridium_XVIII	0.196280137	0.657740317	0.841557071
Coprococcus	2.518172014	0.112540976	0.667784148
Collinsella	11.12049981	0.000853788	0.069156809
Intestinibacter	1.983262986	0.15904708	0.667784148
Lactobacillus	2.703015421	0.10015865	0.667784148
Clostridium_XlVa	1.383827929	0.239450187	0.667784148
Veillonella	0.313663257	0.575440916	0.810621117
Alistipes	0.077906564	0.780154673	0.940668859
Comamonas	0.035093564	0.851399737	0.962761716
Parasutterella	1.552131638	0.212821029	0.667784148
Parabacteroides	0.005661932	0.940019137	0.977056008
Pseudomonas	0.499132708	0.479881451	0.733403727
Megasphaera	1.146006354	0.284386723	0.672793891
Lactococcus	1.941060207	0.163553465	0.667784148
Eubacterium	0.751938312	0.385863243	0.692458852
Oceanobacillus	0.652015387	0.419393046	0.693282383
Haemophilus	2.166689034	0.141029589	0.667784148
Clostridium_XlVb	0.75936561	0.383527126	0.692458852
Terrisporobacter	2.684568156	0.101324691	0.667784148
Akkermansia	0.256444688	0.612573291	0.826973943
Turicibacter	0.000124053	0.991113425	0.991113425
Holdemanella	0.012997131	0.909233862	0.974847057
Fusobacterium	1.901114648	0.167953607	0.667784148
Desulfovibrio	0.004892174	0.944238169	0.977056008
Clostridium_IV	5.729958177	0.01667788	0.315879925
Enterococcus	2.940607032	0.086378266	0.667784148
Paraprevotella	0.00018061	0.989277443	0.991113425
Oscillibacter	0.007146966	0.932627316	0.977056008
Bilophila	0.209809337	0.646916885	0.841557071
Barnesiella	0.072491585	0.787742989	0.940668859
Psychrobacter	0.560898844	0.453898311	0.700300252
Weissella	1.471950371	0.225038221	0.667784148
Flavonifractor	0.742147579	0.388973799	0.692458852
Myroides	0.436220999	0.50895208	0.763428121
Corynebacterium	0.654577079	0.418480992	0.693282383

Saccharibacteria_gene	3.86221545	0.049385077	0.564933626
Actinomyces	4.41575993	0.035608415	0.445302406
Brochothrix	0.693189227	0.40508172	0.692771967
Eggerthella	0.301760154	0.582781061	0.813883895
Acinetobacter	0.571214744	0.449776613	0.700300252
Arthrobacter	0.222772679	0.636935348	0.841557071
Odoribacter	0.262404643	0.608472925	0.826973943
Alloprevotella	0.052522714	0.818730303	0.940668859
Erysipelotrichaceae_in	1.266239521	0.26047333	0.667784148
Butyricimonas	0.067839558	0.79450875	0.940668859
Lysinibacillus	0.38676448	0.53400505	0.768683498
Slackia	1.348759982	0.245495016	0.667784148
Flavobacterium	0.985690099	0.320798038	0.675062408
Providencia	0.146651702	0.70175587	0.854782687
Neisseria	1.412567724	0.234630604	0.667784148
Butyrivibrio	1.227272727	0.267938084	0.667784148
Rothia	0.599232771	0.438870909	0.697030266
Howardella	5.532687664	0.018664414	0.315879925
Allisonella	0.004435591	0.946899959	0.977056008
Morganella	1.562744025	0.211263894	0.667784148
Proteus	0.177397841	0.673619098	0.841557071
Acidaminococcus	1.135711383	0.286560361	0.672793891
Gemella	9.890889462	0.001660991	0.089693494
Solobacterium	1.749523445	0.185936653	0.667784148
Peptostreptococcus	0.405052823	0.524491238	0.765473699
Streptophyta	1.785786543	0.181440375	0.667784148
Leuconostoc	0.984648465	0.321053863	0.675062408
Senegalimassilia	0.269389709	0.603741454	0.826973943
Kocuria	2.49537037	0.114181512	0.667784148
Chryseobacterium	0.246438908	0.61959379	0.829538793
Oribacterium	3.099654988	0.078308888	0.667784148
Holdemania	1.010158127	0.31486496	0.675062408
Carnobacterium	0.020735055	0.885503059	0.962761716
Methylobacterium	0.814814815	0.366700567	0.675062408
Mogibacterium	3.723864126	0.0536401	0.564933626
Christensenella	6.057453563	0.013847722	0.315879925
Coprobacter	0.217478869	0.640967645	0.841557071
Paracoccus	0.677260859	0.410531536	0.692771967
Pyramidobacter	0.027782116	0.867622094	0.962761716
Dolosicoccus	1.227272727	0.267938084	0.667784148
Porphyromonas	0.000457437	0.982936332	0.991113425
Atopobium	5.59207092	0.018041953	0.315879925
Sutterella	2.109901423	0.146348745	0.667784148
Gordonibacter	1.026946859	0.310876843	0.675062408
Janthinobacterium	0.063949606	0.800358941	0.940668859
Abiotrophia	1.811593731	0.178317165	0.667784148
Stenotrophomonas	0.059323734	0.807568017	0.940668859
Sporosarcina	0.956272814	0.32812773	0.675062408
Facklamia	0.146641204	0.701766033	0.854782687
Butyricicoccus	0.677260859	0.410531536	0.692771967
Parvimonas	1.111756264	0.291700494	0.675062408
Coprobacillus	0.002492007	0.960186131	0.981752558
Paenibacillus	0.6945529	0.404620075	0.692771967
Brevundimonas	0.6945529	0.404620075	0.692771967
Anaerofustis	1.136550692	0.286382365	0.672793891
Bhargavaea	1.227272727	0.267938084	0.667784148
Cloacibacillus	0.86139279	0.353349223	0.675062408
Oxalobacter	2.134786873	0.143990473	0.667784148

Anaerotruncus	0.0020859	0.963571955	0.981752558
Brachybacterium	0.185592774	0.666610311	0.841557071
Olsenella	3.658139263	0.055795914	0.564933626
Peptoniphilus	0.382664904	0.536180464	0.768683498
Aerococcus	1.227272727	0.267938084	0.667784148
Lachnoanaerobaculum	0.409896062	0.522022265	0.765473699
Brevibacterium	0.185592774	0.666610311	0.841557071
Pediococcus	1.227272727	0.267938084	0.667784148
Oligella	0.814814815	0.366700567	0.675062408
Staphylococcus	0.626868	0.428506485	0.697030266
Thauera	1.227272727	0.267938084	0.667784148
Acetoanaerobium	NA	NA	NA
Geobacillus	2.49537037	0.114181512	0.667784148
Chishuiella	0.059302921	0.807601113	0.940668859
Achromobacter	NA	NA	NA
Burkholderia	0.586732825	0.443685303	0.697835137
Epilithonimonas	0.175437827	0.675323575	0.841557071
Peptococcus	0.814814815	0.366700567	0.675062408
Aquabacterium	2.495906433	0.114142642	0.667784148
Arcobacter	NA	NA	NA
Atopostipes	0.025725468	0.8725726	0.962761716
Moraxella	NA	NA	NA
Finegoldia	0.48476614	0.486271346	0.736223907
Faecalitalea	0.175437827	0.675323575	0.841557071
Exiguobacterium	2.495191734	0.114194468	0.667784148
Jeotgalicoccus	0.025725468	0.8725726	0.962761716
Sphingobacterium	1.646429935	0.199445533	0.667784148
Shewanella	1.646604938	0.199421648	0.667784148
Aeromonas	0.814814815	0.366700567	0.675062408
Ezakiella	0.814814815	0.366700567	0.675062408
Scardovia	1.327675693	0.249219136	0.667784148
Mobiluncus	0.814814815	0.366700567	0.675062408
Alloscardovia	1.227272727	0.267938084	0.667784148
Anaeroglobus	1.646429935	0.199445533	0.667784148
GpIIa	1.646429935	0.199445533	0.667784148
Helicobacter	1.646604938	0.199421648	0.667784148
Leptotrichia	0.814814815	0.366700567	0.675062408
Ralstonia	0.017221181	0.895593763	0.967241264
Methanobrevibacter	NA	NA	NA
Cellulosilyticum	0.605081486	0.436645751	0.697030266
Ornithinicoccus	1.646429935	0.199445533	0.667784148
Anaerococcus	0.025725468	0.8725726	0.962761716
Nevskia	0.021262977	0.884064828	0.962761716
Delftia	0.814814815	0.366700567	0.675062408
Negativicoccus	0.021262977	0.884064828	0.962761716
Novosphingobium	NA	NA	NA
Reyranella	0.814814815	0.366700567	0.675062408
Campylobacter	1.227272727	0.267938084	0.667784148
Methanosphaera	NA	NA	NA
Mitsuokella	NA	NA	NA
Pseudochrobactrum	0.814814815	0.366700567	0.675062408
Dietzia	0.814814815	0.366700567	0.675062408
Sulfuricurvum	NA	NA	NA
Proteocatella	0.814814815	0.366700567	0.675062408
Caulobacter	NA	NA	NA
Thiopseudomonas	1.227272727	0.267938084	0.667784148
Spartobacteria_genera	NA	NA	NA
Cryptobacterium	NA	NA	NA

Deinococcus	NA	NA	NA
Cupriavidus	1.227272727	0.267938084	0.667784148
Tetragenococcus	NA	NA	NA
Sphingopyxis	NA	NA	NA
Arcanobacterium	NA	NA	NA
GpI	NA	NA	NA
Fusibacter	NA	NA	NA

healthy.median	healthy.1st_Qu.	healthy.3rd_Qu.	ibs.median
12.55	5.6925	22.65	12.8
4.64	0.57	10.625	9.71
8.87	1.66	16.85	7.035
1.78	0.565	17.175	0.815
5.245	2.2775	14.625	3.185
0	0	0.7225	0.02
0.35	0.04	1.13	2.25
0.84	0.0275	2.39	2.385
0.84	0.1975	2.8025	0.92
0	0	0.0225	0
0.125	0.01	0.955	0.495
0.675	0.15	1.66	1.585
0.605	0.0925	3.84	0.995
0.82	0.02	3.8025	0.815
0.255	0.0175	1.7375	0.14
0.005	0	0.1025	0.035
0.04	0.02	0.12	0.03
0.745	0.2625	1.5975	0.785
0.335	0.16	1.1475	0.605
0.29	0.04	0.7275	0.19
0.125	0.0475	0.5	0.205
0.24	0.0375	1.26	0.16
0.17	0.0575	0.44	0.205
0.09	0	0.38	0.195
0.01	0	0.065	0.11
0.16	0.07	0.4725	0.09
0	0	0.02	0.01
0.245	0.055	0.705	0.11
0.02	0	0.0825	0.03
0.035	0	0.4775	0.075
0	0	0	0
0.04	0.0075	0.425	0.03
0.13	0.025	0.3325	0.13
0	0	0.025	0.01
0	0	0	0
0	0	0.03	0.01
0.015	0	0.27	0.07
0	0	0.0125	0.01
0.01	0	0.1025	0.025
0.03	0.0075	0.225	0.065
0.04	0.01	0.0725	0.01
0	0	0	0
0.01	0	0.095	0.01
0	0	0	0
0	0	0.01	0
0	0	0.0025	0
0.025	0.01	0.1025	0.065
0.01	0	0.05	0
0	0	0.045	0
0.02	0	0.0525	0.01
0.01	0	0.07	0.02
0	0	0.02	0
0	0	0	0
0	0	0.01	0
0.01	0	0.0675	0.01
0	0	0	0
0	0	0	0

0.01	0	0.0325	0.025
0.02	0.01	0.04	0.03
0	0	0	0
0	0	0.03	0.005
0	0	0	0
0	0	0	0
0	0	0.01	0
0	0	0	0
0.005	0	0.02	0
0	0	0.03	0
0	0	0	0
0	0	0	0
0	0	0	0
0	0	0	0
0	0	0	0
0	0	0	0
0	0	0.01	0
0	0	0	0
0	0	0	0
0	0	0	0
0	0	0	0
0	0	0	0
0	0	0.0025	0.01
0	0	0.0025	0
0	0	0.01	0
0	0	0	0
0	0	0	0
0	0	0	0
0	0	0	0
0	0	0	0
0	0	0	0
0	0	0	0
0	0	0	0
0	0	0	0
0	0	0	0
0	0	0	0
0	0	0	0
0	0	0	0
0	0	0	0
0	0	0	0
0	0	0	0
0	0	0	0
0	0	0	0
0	0	0	0
0	0	0	0
0	0	0	0
0	0	0	0
0	0	0	0
0	0	0	0
0	0	0	0
0	0	0	0
0	0	0	0
0	0	0	0
0	0	0	0
0	0	0	0
0	0	0	0
0	0	0	0
0	0	0	0

0	0	0	0
0	0	0	0
0	0	0	0
0	0	0	0
0	0	0	0
0	0	0	0
0	0	0	0
0	0	0	0
0	0	0	0
0	0	0	0
0	0	0	0
0	0	0	0
0	0	0	0
0	0	0	0
0	0	0	0
0	0	0	0
0	0	0	0
0	0	0	0
0	0	0	0
0	0	0	0
0	0	0	0
0	0	0	0
0	0	0	0
0	0	0	0
0	0	0	0
0	0	0	0
0	0	0	0
0	0	0	0
0	0	0	0
0	0	0	0
0	0	0	0
0	0	0	0
0	0	0	0
0	0	0	0
0	0	0	0
0	0	0	0
0	0	0	0
0	0	0	0
0	0	0	0
0	0	0	0
0	0	0	0
0	0	0	0
0	0	0	0
0	0	0	0
0	0	0	0
0	0	0	0
0	0	0	0
0	0	0	0
0	0	0	0
0	0	0	0
0	0	0	0
0	0	0	0
0	0	0	0
0	0	0	0
0	0	0	0
0	0	0	0
0	0	0	0
0	0	0	0

0	0	0	0
0	0	0	0
0	0	0	0
0	0	0	0
0	0	0	0
0	0	0	0
0	0	0	0

ibs.1st_Qu.	ibs.3rd_Qu.
7.2375	23.45
3.2525	17.875
2.88	10.8
0.26	4.4725
0.52	6.4175
0	0.545
0.2325	5.5625
0.46	7.2675
0.19	3.065
0	0.03
0.065	2.7175
0.535	3.14
0.19	2.8875
0.2475	1.795
0	1.585
0	0.8075
0.01	0.0875
0.42	2.3925
0.2075	1.6525
0.04	0.8175
0.0825	0.735
0.0225	0.7075
0.04	0.4775
0.0125	0.84
0.0025	0.575
0.02	0.3925
0	0.0675
0.03	0.5475
0.0025	0.105
0	0.32
0	0
0	0.2425
0.0225	0.385
0	0.03
0	0
0	0.0375
0	0.2125
0	0.02
0	0.19
0.01	0.2475
0	0.105
0	0.0075
0	0.115
0	0
0	0
0	0.0075
0.03	0.1375
0	0.01
0	0.025
0	0.0575
0	0.1075
0	0.0175
0	0.01
0	0.01
0	0.03
0	0
0	0

0.01	0.06
0.01	0.06
0	0
0	0.04
0	0
0	0
0	0.01
0	0
0	0.02
0	0.02
0	0
0	0
0	0
0	0
0	0
0	0
0	0.0175
0	0
0	0
0	0
0	0
0	0
0	0.0175
0	0.01
0	0.01
0	0
0	0
0	0
0	0
0	0
0	0.01
0	0.01
0	0
0	0
0	0
0	0.0075
0	0
0	0
0	0
0	0
0	0
0	0.0075
0	0
0	0
0	0
0	0
0	0
0	0
0	0
0	0
0	0
0	0
0	0
0	0
0	0
0	0
0	0
0	0

0	0
0	0
0	0
0	0
0	0
0	0
0	0
0	0
0	0
0	0
0	0
0	0
0	0
0	0
0	0
0	0
0	0
0	0
0	0
0	0
0	0
0	0
0	0
0	0
0	0
0	0
0	0
0	0
0	0
0	0
0	0
0	0
0	0
0	0
0	0
0	0
0	0
0	0
0	0
0	0
0	0
0	0
0	0
0	0
0	0
0	0
0	0
0	0
0	0
0	0
0	0
0	0
0	0
0	0
0	0
0	0
0	0
0	0

0	0
0	0
0	0
0	0
0	0
0	0
0	0
